# Comparison of the Proteome of Huh7 Cells Transfected with Hepatitis B Virus Subgenotype A1, with or without G1862T

**DOI:** 10.3390/cimb46070419

**Published:** 2024-07-04

**Authors:** Kiyasha Padarath, Aurélie Deroubaix, Previn Naicker, Stoyan Stoychev, Anna Kramvis

**Affiliations:** 1 Hepatitis Virus Diversity Unit, Department of Internal Medicine, School of Clinical Medicine, Faculty of Health Science, University of Witwatersrand, 7 York Road, Johannesburg 2193, South Africa; 2 Life Sciences Imaging Facility, Faculty of Health Sciences, University of the Witwatersrand, 7 York Road, Johannesburg 2193, South Africa; 3 Future Production Chemicals, Council for Scientific and Industrial Research, Pretoria 0001, South Africa; pnaicker1@csir.co.za; 4 ReSyn Biosciences, Johannesburg 2194, South Africa; sstoychev@resynbio.com; 5 Evosep Biosystems, 5230 Odense, Denmark

**Keywords:** G1862T, HBV, subgenotype A1, p38 MAPK, replication protein A (RPA), DNA primase (PRIM2)

## Abstract

HBeAg is a non-structural, secreted protein of hepatitis B virus (HBV). Its p25 precursor is post-translationally modified in the endoplasmic reticulum. The G1862T precore mutation leads to the accumulation of P25 in the endoplasmic reticulum and activation of unfolded protein response. Using mass spectrometry, comparative proteome profiling of Huh-7 cells transfected with wildtype (WT) or G1862T revealed significantly differentially expressed proteins resulting in 12 dysregulated pathways unique to WT-transfected cells and 7 shared between cells transfected with either WT or G1862T. Except for the p38 MAPK signalling pathway, WT showed a higher number of DEPs than G1862T-transfected cells in all remaining six shared pathways. Two signalling pathways: oxidative stress and cell cycle signalling were differentially expressed only in cells transfected with G1862T. Fifteen pathways were dysregulated in G1862T-transfected cells compared to WT. The 15 dysregulated pathways were involved in the following processes: MAPK signalling, DNA synthesis and methylation, and extracellular matrix organization. Moreover, proteins involved in DNA synthesis signalling (replication protein A (RPA) and DNA primase (PRIM2)) were significantly upregulated in G1862T compared to WT. This upregulation was confirmed by mRNA quantification of both genes and immunofluorescent confocal microscopy for RPA only. The dysregulation of the pathways involved in these processes may lead to immune evasion, persistence, and uncontrolled proliferation, which are hallmarks of cancer.

## 1. Introduction

In Africa, there are an estimated 64.7 million people chronically infected with hepatitis B virus (HBV). This is an underestimate due to inadequate screening and disease surveillance [1]. During chronic hepatitis B (CHB), hepatitis B e antigen (HBeAg) is used as a biomarker for viral infection and patient outcomes [2,3]. HBeAg is a non-structural protein, that has a dual role as a tolerogen and immunogen [4]. The loss of HBeAg with reduced viral replication is considered a favourable sign for patients with CHB [2,5]. HBeAg loss associated with persistently elevated HBV DNA levels can lead to cirrhosis and hepatocellular carcinoma (HCC) [6]. Therefore, HBeAg-negative CHB represents a clinical challenge [7,8].

Several basic core promoter (BCP) and precore (PC) gene mutations in HBV can affect HBeAg expression leading to HBeAg-negative CHB [9,10]. In southeastern sub-Saharan Africa (SSA), the predominant subgenotype is A1. Subgenotype A1 has a 4.5-fold higher risk of developing HCC at an earlier age than other HBV genotypes [11]. Subgenotype A1 has unique BCP/PC mutations that affect the expression of HBeAg at a transcriptional, translational, and post-translational level [12]. In particular, the G1862T mutation can be found in HBV isolated from both tumour and non-tumorous liver tissues but is more prevalent in tumorous tissue [13]. G1862T is a missense mutation resulting in a G to T transversion at amino acid 18 of precore precursor p25, which converts valine to phenylalanine and results in a bulky aromatic ring that interferes with the signal peptide cleavage of p25, between positions 18 and 19 [13]. Therefore, G1862T leads to the accumulation of the HBeAg precursor (p25) in the cytoplasm of the hepatocyte and decreased secretion of mature HBeAg [14,15,16].

Previous studies have shown that G1862T mutant precursor accumulates in the endoplasmic reticulum (ER), endoplasmic reticulum–Golgi intermediate compartment (ERGIC) and Golgi [16,17,18]. These aggregates of mutant protein attract ubiquitin, heat shock proteins and proteasomes and cause ER stress and the earlier activation of the unfolded protein response (UPR) cascade [17]. As the ER is a quality-control organelle for proteostasis, this accumulation of proteins in the ER, ER stress and the activation of UPR can disturb proteostasis, affecting host signalling pathways involved in epigenetics, mitochondrial regulation, autophagy, and metabolism [19]. Furthermore, an increase in ER stress can result in liver damage, which is a contributing factor to hepatocarcinogenesis and may explain why G1862T is frequently found in HCC patients compared to asymptomatic carriers of HBV [12].

Proteomic studies have been used to describe global changes in liver cells from patients with and without HBV-associated liver disease [20,21,22]. Lu et al. found dysregulated proteins in plasma from patients with fibrosis compared to healthy controls [23]. Dysregulation of the proteome was also found in tumorous compared to adjacent non-tumorous tissues when liver biopsies from HCC patients were compared [20]. When serum from CHB patients infected with genotype B or C and healthy controls were compared to each other, proteomic analysis revealed dysregulation of the expression of various proteins [22]. Proteome alterations involved in different biological processes included translation [20,23], oxidation reduction [20,21,22,23], intracellular transport [22,23], establishment of protein localization [22,23], protein transport [21,22,23], and translational elongation [20,21,22].

The objective of this study was to use mass spectrometry to determine the effect of G1862T on the proteome, by transfecting Huh7 cells with replication-competent clones of subgenotype A1, with or without G1862T. Having previously shown the accumulation of HBeAg in the ERGIC and the upregulation of the UPR [14], this in vitro system was used to determine the downstream effects of G1862T on host signalling pathways. Little is known about the difference in specific biological pathways induced by the accumulation of mutant HBeAg in the ER. Understanding the role of G1862T mutation may provide further insights on the pathogenesis of HCC in SSA patients infected with subgenotype A1.

## 2. Materials and Methods

### 2.1. Plasmid Constructs

1.28-mer replication-competent plasmids for wild-type subgenotype A1 (KM519453) and subgenotype A1 with the G1862T mutation (KM519452) were constructed and were able to replicate and express proteins in vitro [24,25]. Ethics approval to perform this study was obtained from the University of the Witwatersrand Research Ethics Committee (Medical), Johannesburg, South Africa (HREC: W-CBP-210416-01).

### 2.2. Cell Line and Transfections

Huh7 cells were transfected as described previously [24]. Twenty-four hours before transfection, 3 × 10^6^ Huh7 cells were plated into each of the 10 cm culture dishes for mass spectrometry, whilst 2 × 10^5^ Huh7 cells were plated in each of 12-well plates either with coverslips for indirect immunofluorescence or no coverslips for real-time polymerase chain reaction (RT-PCR) (viral load/RNA expression) or enzyme-linked immunosorbent assay ELISA and incubated overnight at 37 °C in a humidified incubator containing 5% (*w*/*v*) CO_2_. For the transfection, 12,000 ng/10 cm-plate and 800 ng for each well of a 12-well plate were used. All experiments were carried out using 4 replicates per condition. To measure transfection efficiency 1-day post-transfection, an eGFP control was included in triplicate. This was measured using The FLoid™ Cell Imaging Station (Thermo Fisher, Waltham, MA, USA) by counting the number of cells successfully transfected.

### 2.3. Mass Spectrometry Analysis

Mass spectrometry was performed using the protocol described by Padarath et al. [26].

#### 2.3.1. Cell Lysis and Protein Preparation

Five days post-transfection, cells were washed 3 times with PBS. Aliquots of 0.5 million cells were prepared and pelleted at 300× *g* for 10 min. Supernatants were removed and cell pellets were stored at −80 °C until further use. Cell pellets were thawed on ice and resuspended in 200 μL of lysis buffer (1% SDS, 50 mM Tris-HCl pH 8.0). Protein was extracted from cell pellets using a PIXUL multi-sample sonicator (Active Motif, Carlsbad, CA, USA). Sonication settings were: Pulse = 50; PRF = 1; Process Time = 30; Burst = 20. Thereafter, the lysates were incubated with 25 units of benzonase, and with MgCl_2_ to a final concentration of 2 mM at 37 °C for 30 min. Next, the samples were centrifuged at 15,000× *g* to clear cell debris. Post BCA-based protein concentration determination, samples were incubated with 10 mM DTT (30 min at 37 °C) followed by addition of 40 mM IAA (30 min at RT).

#### 2.3.2. Sample Clean-Up and Digestion

All experiments were performed with a KingFisher™ Flex (Thermo Fisher Scientific, Waltham, MA, USA) magnetic particle processing robot, as previously described [27]. The KingFisher™ Flex system was configured for automated HILIC-protein clean-up and on-bead trypsin digest. In brief, deep 96 well plates were loaded in each carousel position with each plate filled as follows: (1) 96 well tip heads (Thermo Fisher Scientific, Waltham, MA, USA); (2) 10 µL, 20 mg/mL hyper porous magnetic MagReSyn HILIC microspheres (ReSyn Biosciences, Pretoria, South Africa) in 20% EtOH and 180 µL Equilibration buffer (100 mM NH_4_Ac, 15% MeCN pH 4.5); (3) Equilibration Buffer (500 µL); (4) Protein extract mixed 1:1 with Bind buffer (200 mM NH_4_Ac, 30% MeCN pH 4.5), the final volume of 100 µL; (5) 500 µL 95% MeCN (wash 1); (6) 500 µL 95% MeCN (wash 2); (7) 200 µL 50 mM ammonium bicarbonate pH 8.0 and Promega sequencing grade Trypsin for an enzyme: protein ratio of 1:10; (8) Elution with 100 µl of 1% TFA. The Bindit programme was then run with the magnetic pins transferring the magnetic HILIC beads from position 2 to 8 and in the process binding proteins, washing off SDS and other contaminants and finally generating peptides ready for LC-MS analysis post the on-bead trypsin digest.

#### 2.3.3. LC-MS Data Acquisition

Post-HILIC, peptide samples were vacuum dried and resuspended in 2% MeCN/0.2% FA. Analysis was performed on a Dionex Ultimate 3000 RSLC system coupled to an AB Sciex 5600 TripleTOF mass spectrometer (Sciex, Toronto, ON, Canada). Injected peptides were inline de-salted using an Acclaim PepMap C18 trap column (Thermo Fisher, Waltham, MA, USA) (75 μm × 2 cm; 2 min at 5 μL.min^−1^ using 2% ACN/0.2% FA). Trapped peptides were gradient eluted and separated on a Waters Acquity CSH C18 NanoEase column (Waters^TM^, Milford, MA, USA) (75 μm × 25 cm, 1.7 µm particle size) at a flow-rate of 0.3 µL.min^−1^ with a gradient of 6–40% B over 60 min (A: 0.1% FA; B: 80% ACN/0.1% FA). For Sequential window acquisition of all theoretical mass spectra (SWATH), precursor scans were acquired from 400 to 1100 *m*/*z* with 50 milliseconds accumulation time and fragment ions acquired from 200 to 1800 *m*/*z* for 48 variable-width precursor windows with 0.5 Da overlap between windows and 20 milliseconds accumulation time per window.

#### 2.3.4. SWATH-MS Data Processing

SWATH data was processed using Spectronaut v17 software (Biognosys, Zurich, Switzerland). The default directDIA identification and quantification settings were used for data processing. Carbamidomethylation was added as a fixed modification, and N-terminal acetylation and methionine oxidation were added as variable modifications. Swiss-Prot Human sequences (www.uniprot.org, accessed on 12 December 2022) and common contaminating proteins were used as the search databases. A q-value ≤ 0.01 cut-off was applied at the precursor and protein levels. Quantification was performed at the MS1 and MS2 level. Label-free cross-run normalisation was employed using a global normalisation strategy. Candidate dysregulated proteins were filtered at a q-value ≤ 0.05, absolute fold change (FC) ≥ 1.5 and a minimum of two unique peptides identified.

#### 2.3.5. Mass Spectrometry Biological Data Annotation

Pathway analysis, gene-set enrichment analysis, and STRING analysis were performed as described in Padarath et al. [26].

### 2.4. RNA Expression Levels

As described in Padarath et al. [26], total RNA was isolated from Huh7 cells-transfected with wildtype subgenotype A1, subgenotype A1 with G1862T mutation, and controls (untransfected and vector controls) using the RNAeasy mini extraction kit (Qiagen, Hilden, Germany), according to the manufacturer’s instructions. An aliquot of 500 ng RNA was reverse transcribed to generate cDNA using the SuperScript™ VI cDNA First-Strand Synthesis System synthesis kit (Invitrogen, Boston, MA, USA), as per the manufacturer’s instructions. A total of 5 µL of cDNA (1:10 dilution) was amplified using SYBR^®^Green Realtime PCR Master Mix (Thermofisher, Waltham, MA, USA) and performed on a MyCycler Thermocycler (Bio-Rad Laboratories, Hercules, CA, USA). Thermal cycling conditions were as follows: initial denaturation at 95 °C for 2 min followed by 25 cycles of denaturation at 95 °C for 30 s, annealing at 55 °C for 30 s, elongation at 72 °C for 1 min, and a final elongation at 72 °C for 10 min. Three relative expression levels of the target genes were calculated using the ∆Ct method with GAPDH as the control housekeeping gene. Primers for the qPCR are listed in Table 1 below.

### 2.5. Viral Loads

Viral loads were performed using the protocol described by Bhoola et al. [24]. On day 5 post-transfection, total DNA was extracted from transfected Huh7 cells harvested using the NucleoSpin^®^ Tissue kit (Macherey-Nagel, Nordrhein-Westfalen, Düren, Germany). An aliquot of 5 mL of transfected medium collected on day 3 or 5 was centrifuged at 22,000× *g* for 5 min at 4 °C, after which a 100 μL aliquot was adjusted with 10 μL 10× Incubation Buffer (Roche Diagnostics, Basel, Switzerland) and treated with 1 µL 10 U/µL DNase I (Roche Diagnostics) and 10 µL 100 mg/mL RNase A (Fermentas Molecular Biology Tools, Waltham, MA, USA) and incubated at 37 °C for 20 min. Subsequently, the reaction was terminated by adding 5 µL 0.2 M EDTA, pH 8.0 and incubation at 75 °C for 10 min. Thereafter, extracellular total DNA and intracellular total DNA were extracted using the NucleoSpin^®^ Tissue. PCR primers, HBV DNA F (5′-CGTGTGTCTTGGCCAAAATTCG-3′) and HBV DNA R (5′-CATCCAGCGATAACCAGGACAA-3′), with a FAM/NFQ, labelled TaqMan^®^ MGB probe (5′-FAM-TCACTCACCAACCTCC-NFQ-3′) were used to quantify HBV DNA in the ABI 7500 Real-Time PCR system (Applied Biosystems by Life Technologies, Waltham, MA, USA) using the PCR thermal cycling conditions described previously [28]. A serial dilution of plasmid DNA containing a single genome of subgenotype A1, ranging from 1 × 10^4^ to 1 × 10^8^ copies/mL, was used as a template to generate the standard curve. The 2nd WHO International Standard, Hepatitis B Virus DNA (National Institute for Biological Standards and Controls (NIBSC), Hertfordshire, UK), which has a final concentration of 1 × 10^6^ IU/mL was used as the positive control and to calibrate the standard curve.

### 2.6. HBeAg Expression

Expression of HBeAg in the culture media (supernatants) was analysed with an ELISA kit (ELISA kit: ETI-EBK PLUS (HBeAg), (DiaSorin, Saluggia, Italy) according to the manufacturer’s protocol. The concentrations were determined in OD/µL for the transfection of each construct and compared to the control provided in the kit.

### 2.7. Immunofluorescence

Protocols were adapted from Deroubaix et al. [18]. Cells cultured in 12-well plates onto coverslips were washed 3 times with PBS and fixed with 3.7% formaldehyde in 1× PBS for 10 min at room temperature. The fixed cells were washed 3 times again with 1× PBS and permeabilized with 0.1% Triton-PBS for 7 min and washed 3 times with PBS. Cells were incubated for 1 h at room temperature with 1% bovine serum albumin (BSA, Fraction V, Roche Diagnostics GmbH by Roche Applied Science, Basel, Switzerland) (diluted in PBS 1×). Next, the cells were incubated with the primary antibodies (transfected cells: rabbit anti-HBV core protein DAKO [29], Agilent Technologies (Santa Clara, CA, USA), 1/1000, mouse anti-RPA, Invitrogen, 1/200) for 1 h at 37 °C. Then, the cells were washed 8 times with PBS 1× and incubated with the secondary antibody (Alexa Fluor 488/labelled anti-mouse (1:1000, Invitrogen) and Alexa Fluor 546 labelled anti-rabbit (1:1000, Invitrogen) for 1 h at 37 °C. Cells were washed 8 times with PBS 1×. DNA was stained with 4′,6-Diamidino-2-Phenylindole, Dihydrochloride (DAPI, 1 mg/mL, 1/2000, Sigma-Aldrich, Saint Louis, MO, USA) for 10 min at room temperature in the dark. Coverslips were mounted using Fluoromount™ Aqueous Mounting Medium (Sigma-Aldrich), sealed using clear nail polish after 3 h and analysed by fluorescent microscopy the following day.

### 2.8. Microscopy and Image Analysis

Microscopy was performed using a Zeiss Laser Scanning Confocal Microscope 780 (Carl Zeiss AG, Oberkochen, Germany), equipped with a 63× objective (oil immersion alpha Plan-Apochromat 63×/1.40 Oil CorrM27 (Zeiss)) and Zen Blue software 2.1. Quantification of fluorescence was performed using ImageJ software (Version 1.53j, Fiji/Image J, https://imagej.net/Fiji/Downloads, accessed on 29 June 2023), as described by Deroubaix et al. [18]. Briefly, the mean of replication protein A (RPA) fluorescence in the nucleus of 20 transfected cells was determined per condition and compared to the untransfected control. Laser intensity (488 nm laser for Alexa Fluor 488, 546 nm for Alexa 546 and 405 nm laser for DAPI), was kept constant to obtain comparable results between different slides of an experiment. Using immunofluorescence, a range of 58–68%/field of cells transfected with either WT or G1862T were stained positively for viral antigens (HBcAg and HBeAg) using anti-HBc (Dako, Santa Barbara, CA, USA).

### 2.9. Data Statistical Analysis

Statistical tests, i.e., *t*-test, one-way ANOVA and two-way ANOVA, were calculated using GraphPad Prism version 10.2.2.

## 3. Results

### 3.1. Comparison of Viral Load and HBeAg Expression

Cells transfected with G1862T mutant, showed a significant decrease in extracellular levels of HBeAg when compared to cells transfected with wildtype A1 (Figure S1A). Five days post-transfection, there was no significant difference between the intracellular and extracellular viral loads of cells transfected with either of the two constructs (Figure S1B).

### 3.2. Comparison of the Proteome of Huh7 Cells Transfected with Wildtype and Mutant Subgenotype A1 Compared to the Vector Control

The proteome of cells transfected with one of the following: wildtype, G1862T mutant or vector control, was analysed. At 1% false discovery rate (FDR), 3906 proteins and 36,107 peptides were identified, five days post-transfection. As an internal validation, principal component analysis (PCA) was used to differentiate the proteome of untransfected cells relative to cells transfected with one of the following: vector control, wildtype (WT) and G1862T mutant (Figure S2A). Principal component analysis (PCA) differentiated the untransfected control, vector control, wildtype and G1862T mutant sample groups.

**Figure 1:**
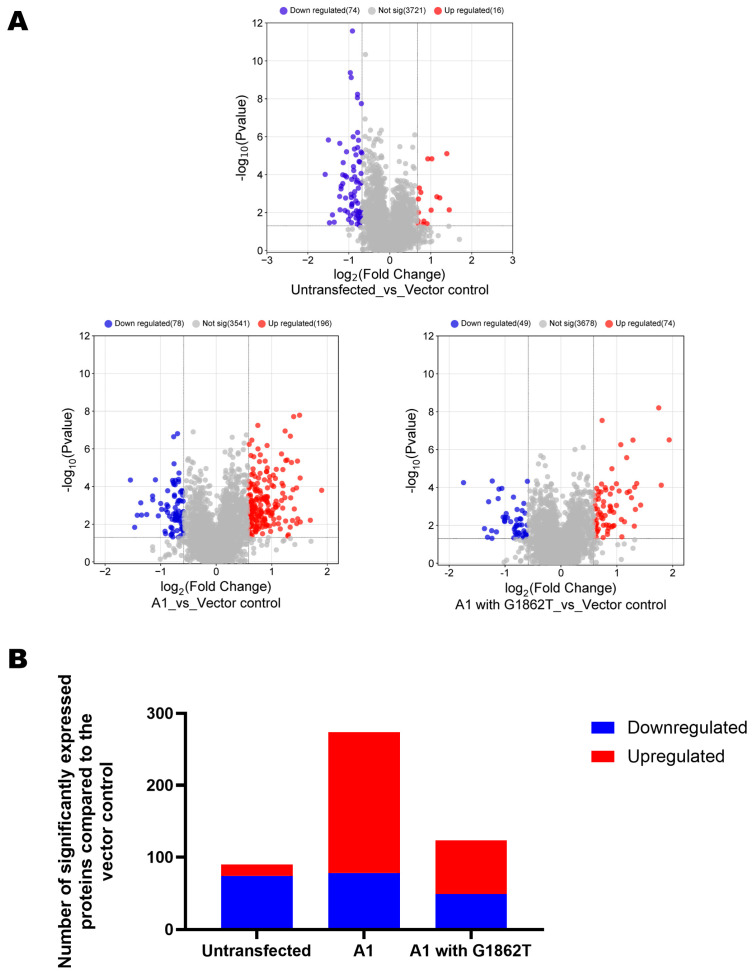
DEPs detection. (**A**) Volcano plots of the differentially expressed proteins, in Huh7 cells, transfected with replication-competent A1 with or without G1862T mutation and untransfected control against the vector-only control. Downregulated versus upregulated proteins are highlighted in blue and red, respectively. (**B**) A bar graph showing the number of significantly upregulated (red) and downregulated (blue) proteins in subgenotype A1, with and without the G1862T mutation and the untransfected compared to the vector control.

Volcano plots were generated to visualize differentially expressed proteins (DEPs) in transfected and untransfected cells (Figure 1A). A minimum fold change ≥1.5 and maximum FDR adjusted *p*-value (q-value) ≤ 0.05 was used to filter proteins that were significantly different between the cells transfected with either WT or G1862T against the vector control. Relative to the untransfected cells, the vector control showed 90 DEPs, 16 downregulated and 74 upregulated. To compensate for the effect of the vector on the proteome of Huh7 cells and of transfection, all comparisons of the proteome of transfection with HBV plasmid constructs were carried out against cells transfected with vector alone (vector control). Thus, relative to the vector control, cells transfected with WT had 247 DEPs (196 upregulated and 78 downregulated), whereas cells transfected with G1862T had 123 DEPs (74 upregulated and 49 downregulated) (Figure 1B).

**Figure 2:**
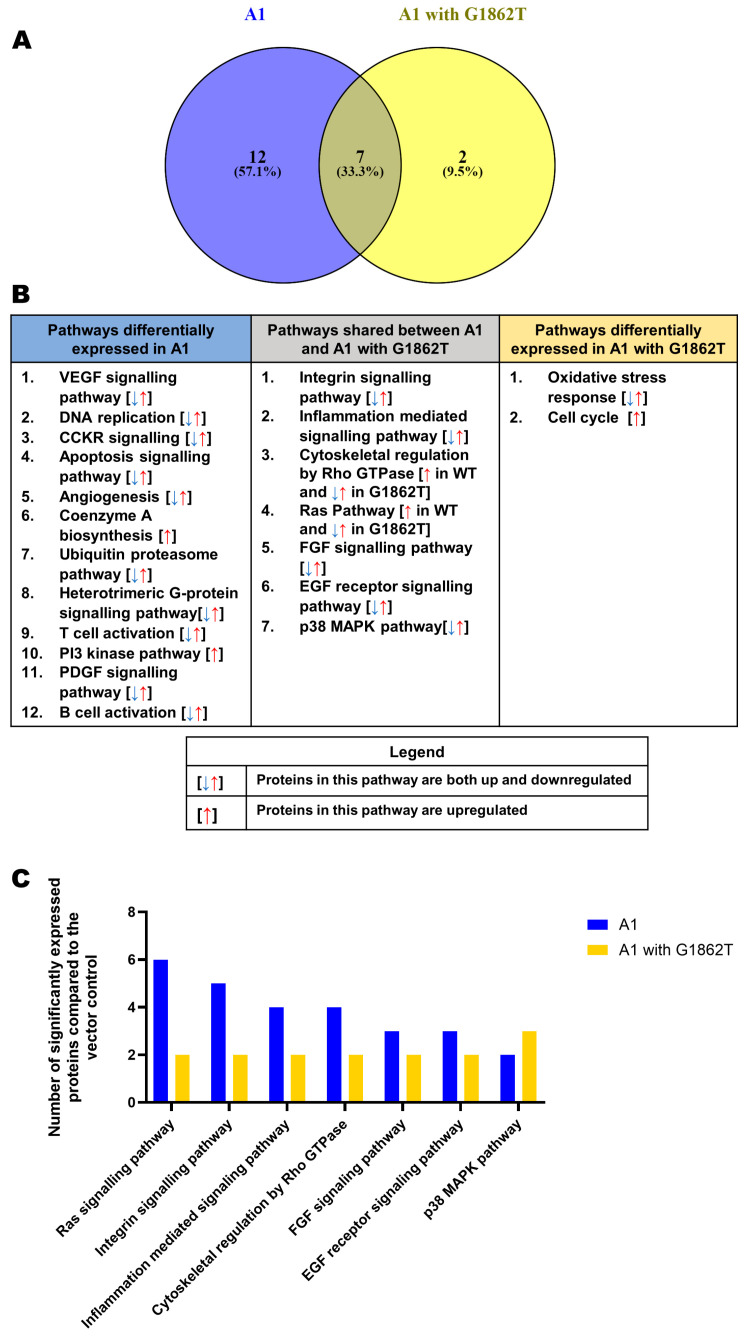
Proteomic analysis. (**A**) Venn diagram indicating the pathways associated with differentially expressed pathways between A1 with (yellow) and without (blue) the G1862T. Proteins were normalized against both the untransfected and vector control. (**B**) Proteomic analysis revealed significantly differently expressed proteins (*p* < 0.05) between the A1 with and without the G1862T. These differentially expressed proteins were further classified into pathways displayed in the table. Blue pathways are exclusively found in A1, grey pathways are shared between A1 with and without G1862T, and yellow pathways are exclusively found in A1 with G1862T. (**C**) Comparison table of the number of significantly differentially expressed proteins, amongst the common pathways between A1 with and without G1862T.

For clarification, DEPs were classified according to their corresponding pathways using the Panther bioinformatics tool, accessed 23 March 2023. Pathways were ranked according to the number of DEPs, and pathways with fewer than two DEPs were excluded from the analysis (Figure 2). In comparison to the vector control, 12 pathways were unique to WT-transfected cells, 2 were unique to G1862T-transfected cells and 7 were shared between cells transfected with either WT or G1862T (Figure 2A). In cells transfected with WT, 12 pathways were differentially expressed. The expression of the proteins in the 10 pathways were either up or downregulated, whereas in the coenzyme A biosynthesis and PI3 kinase pathways the proteins were upregulated (Figure 2B). The seven shared pathways were integrin signalling, inflammation-mediated signalling, cytoskeletal regulation by Rho GTPases, rat sarcoma (Ras) signalling, fibroblast growth factor (FGF) signalling, epidermal growth factor (EGF) signalling and the p38 MAPK signalling pathways (Figure 2B). The dysregulation of five of these pathways was the same for WT and G1862T. On the other hand, cytoskeletal regulation by Rho GTPases and the Ras pathway were upregulated in cells transfected with WT and dysregulated in G1862T-transfected cells. Two signalling pathways, oxidative stress and cell cycle signalling, were differentially expressed only in cells transfected with G1862T, with the former and latter pathways, dysregulated and upregulated, respectively (Figure 2B). Except for the p38 MAPK signalling pathway, WT-transfected cells showed higher DEPs than G1862T-transfected cells in all remaining six shared pathways (Figure 2C).

### 3.3. Proteomic Analysis of Huh7 Cells Transfected with Wildtype Compared to the Mutant

**Figure 3:**
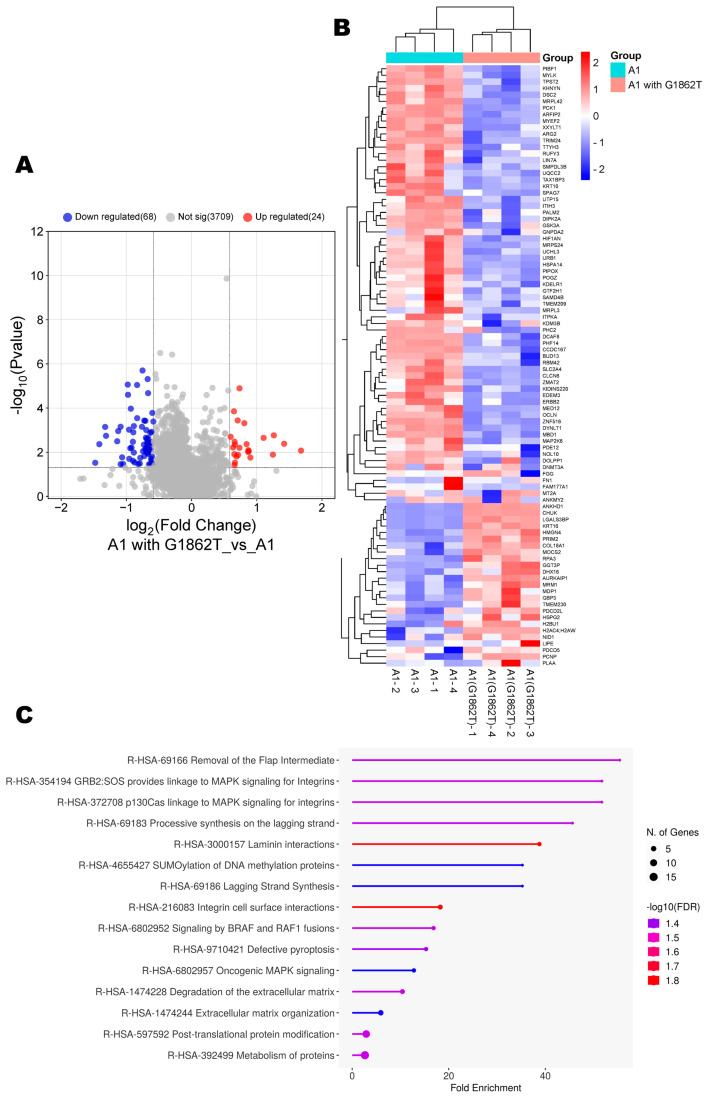
A1 with G1862T vs. A1 analysis. (**A**) Volcano plots of the differentially expressed proteins in Huh7 cells, transfected with replication-competent A1 with or without G1862T mutation. The negative x-axis represents downregulated (blue) in the A1 mutant, and the positive axis represents upregulated (red) proteins in the A1 mutant. (**B**) The heatmap showing the average log2 expression of the 92 significantly expressed proteins in G1862T compared to the WT. (**C**) A dot plot generated using the DEPs from image B in ShinyGO analysis, with Reactome pathway enrichment and fold enrichment, based on the number of genes present in each pathway. The FDR cut-off was set at 0.05, and the number of pathways was set to 10.

Next, the proteome of Huh7 cells transfected with G1862T was compared to WT-transfected cells. Compared to the WT, cells transfected with G1862T had 92 DEPs (24 upregulated and 68 downregulated) (Figure 3A,B). ShinyGO Gene Ontology enrichment analysis [30] was performed on the DEPs. In total, 15 pathways were dysregulated in G1862T-transfected cells compared to those transfected with WT. The 15 dysregulated pathways were involved in the following processes: MAPK signalling, DNA synthesis and methylation, and extracellular matrix organisation (Figure 3C).

The proteome of G1862T-transfected cells was analysed by using Gene Set Enrichment Analysis (GSEA) [31]. The significantly enriched (upregulated) or depleted (downregulated) genes were classified according to their Reactome pathway. The top five GSEA-upregulated pathways were: RNA polymerase II transcription pre-initiation and promoter opening, transcriptional regulation by RUX1, base excision repair and apoptotic cleavage of cellular proteins pathways, and detoxification of reactive oxygen species (Figure S3A). The top five GSEA-downregulated pathways were: rRNA modification in the nucleus and cytosol, mitochondrial tRNA aminoacylation, translocation of SLC2A4-GLUT4 to the plasma membrane, RAF activation and phosphatidylinositol (PI) metabolism (Figure S4A). STRING analysis showed some interconnection between several upregulated and downregulated proteins (Figures S3B and S4B).

### 3.4. Increased Transcription of DNA Primase Subunit 2 (PRIM2) and RPA in G1862T-Transfected Cells Compared to WT-Transfected Cells

Using several proteomic bioinformatic tools, enrichment was observed in proteins relating to DNA synthesis. Amongst the 28 upregulated proteins, 2 proteins of interest were identified: DNA primase subunit 2 (PRIM2) and RPA for their involvement in DNA synthesis and their oncogenic association [32]. Both proteins were significantly upregulated (>2.0-fold change increase) in cells transfected with G1862T compared to WT (Figure 3B).

**Figure 4:**
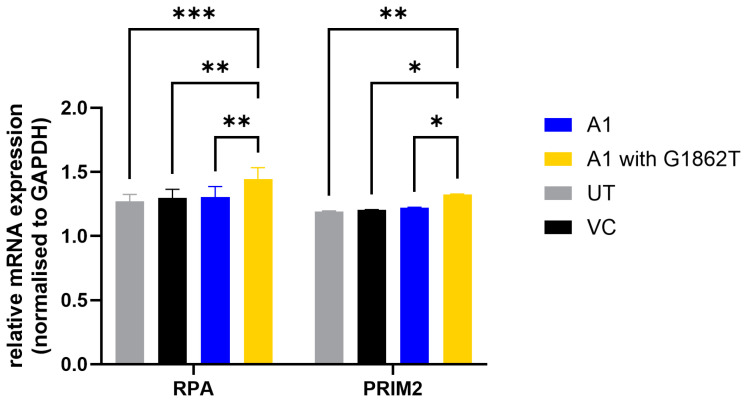
mRNA expression levels of DNA primase subunit 2 (PRIM2) and Replication Protein A (RPA) genes five days post-transfection. Results are expressed as a ratio between the mRNA expression of the DNA synthesis-associated proteins and GAPDH. Statistical analysis was performed using a one-way ANOVA statistical test (* = *p*-value < 0.05, ** = *p*-value < 0.01, *** = *p*-value < 0.001).

Increased transcription of both PRIM2 and RPA genes was shown using RT-qPCR. PRIM2 and RPA mRNA levels were significantly higher in G1862T-transfected cells compared to WT-transfected cells (Figure 4).

### 3.5. Immunofluorescence Reveals Increased Expression of RPA in G1862T-Transfected Cells Compared to WT-Transfected Cells

**Figure 5:**
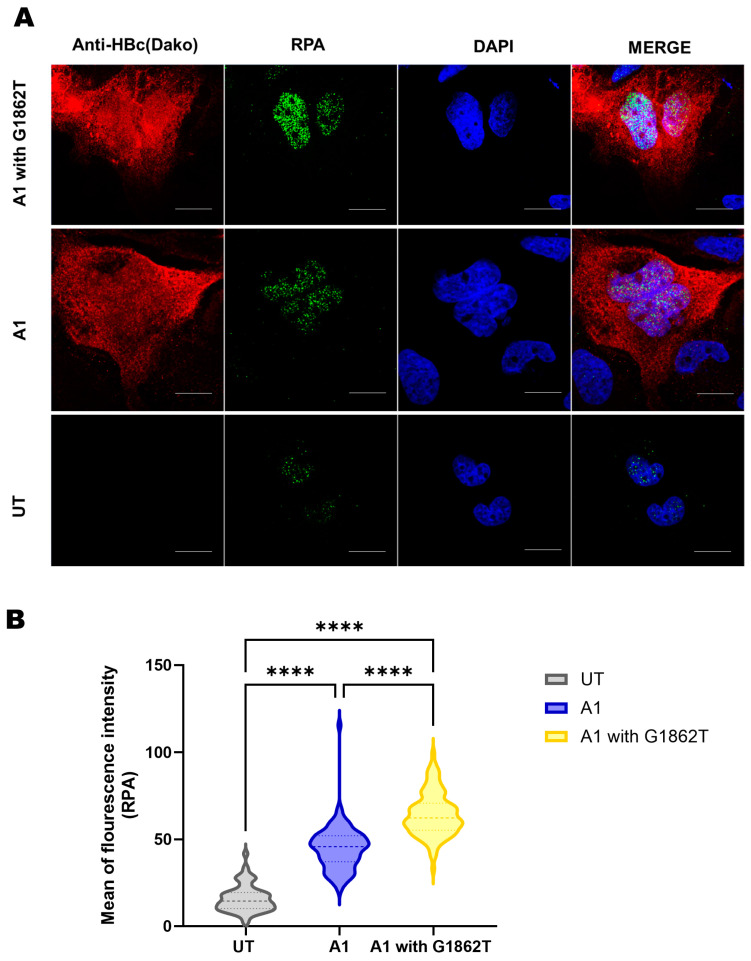
RPA expression using confocal microscopy. (**A**) Microscopy of transfected Huh7 cells with or without G1862T and untransfected cells after five days, viewed with a confocal microscope. Cells were immunostained with a polyclonal rabbit anti-HBc antibody (DAKO) and monoclonal mouse anti-RPA antibody. The nuclei were visualised by DAPI staining. Scale bars = 15 µm. (**B**) A volcano plot indicating the mean of fluorescence intensity for the RPA expression in cells transfected with and without G1862T compared to the untransfected (UT) control. Significant differences were analysed using a one-way ANOVA statistical test and compared to the expression in subgenotype A1 (**** = *p*-value < 0.0001).

For further confirmation, immunofluorescence using the RPA-specific antibody was performed. Confocal microscopy showed that the mean of fluorescent intensity was significantly higher in the nuclei of G1862T-transfected cells compared to the WT-transfected cells and the untransfected control (Figure 5A,B). It should be noted the anti-HBc (Dako) was used to detect HBV proteins (HBcAg and HBeAg), and therefore, this antibody does not differentiate between core and precore proteins.

## 4. Discussion

In agreement with previous studies performed by our team and others [16,17,18,33], HBeAg expression was reduced in cells transfected with G1862T precore mutation (Figure S1A). Viral loads did not differ between cells transfected with WT compared to those transfected with G1862T mutant (Figure S1B). Thus, any difference in protein expression cannot be attributed to differences in viral load.

Using a DIA SWATH-MS-based quantitative proteomic approach, a comparative analysis of the whole proteome of Huh7 cells transfected with either WT or G1862T was performed to determine the effect of G1862T on host signalling pathways. Compared to the vector control, G1862T-transfected cells showed a decrease in the number of significantly expressed proteins compared to WT-transfected cells (Figure 1B). The introduction of G1862T leads to the accumulation of the precursor of HBeAg p25 in the ERGIC [16,17,18,33]. This accumulation causes ER stress and the dysregulation of the UPR cascade [17] and can interfere with proteostasis, which regulates a balanced, functional proteome [19]. In fact, the accumulation of HBsAg in the ER has been shown to affect the expression of host proteins [34]. Moreover, the cell’s transcriptional and translational programmes are also altered in order to overcome the ER stress and resolve protein-folding defects [35,36,37,38]. These consequences may account for the reduced number of significantly expressed proteins observed in cells transfected with the G1862T.

Proteomic analysis revealed that there were several common pathways shared between G1862T- and WT-transfected cells (Figure 2B). The p38 MAPK pathway was the only shared pathway where G1862T-transfected cells had more differentially expressed proteins than WT-transfected cells (Figure 2C). ShinyGo analysis further supported this finding with the enrichment in oncogenic MAPK signalling when G1862T was compared to WT (Figure 3C). p38 MAPK signalling has been shown to play an important role in immune escape as well as HBV replication, the promotion of tumour cell survival and drug resistance [39,40,41]. Previous studies have shown that HBeAg suppresses p38 MAPK phosphorylation [42]. This downregulation of MAPK would allow HBV to evade immune surveillance and thus persist. Therefore, viral mutations such as G1862T, which reduce HBeAg expression, may lead to the removal of the tolerogenic effect of HBeAg and the activation of the immune response [4,6,12].

Furthermore, proteomic analysis revealed that proteins involved in the cell cycle and oxidative stress pathways were exclusively found in cells transfected with the G1862T compared to WT (Figure 2B). This result was further supported by the GSEA analysis, which showed the base excision repair and detoxification of reactive oxygen species pathways were part of the top five enriched pathways when G1862T was compared to WT (Figure S3A). Oxidative stress is increased in HBV infections [43] and can be further exacerbated by the ER stress caused by G1862T [17] through the production of reactive oxygen species from the UPR [44]. Furthermore, increased oxidative stress in HBV infection was also demonstrated to increase DNA damage [45,46]. Therefore, there seems to be an interplay between these two exclusive pathways, which could contribute to the hepatocarcinogenic potential of HBV with G1862T. In addition, it has been shown that p22 and p25, which accumulate in the cytoplasm of G1862T-transfected cells, can translocate to the nucleus [47]. In the nucleus, these HBeAg precursors may interact with the host genome leading to genomic instability and mutations, a known hallmark of cancer [48].

Defective and inappropriate repair of DNA damage can result in genomic instability and promote tumorigenesis [49]. Therefore, the DNA synthesis and repair pathways were further explored. DNA primase subunit 2 (PRIM2) is a subunit of the DNA primase complex and a component of the DNA polymerase alpha complex, which plays an essential role in the initiation of DNA synthesis during the S-phase of the cell cycle [50,51,52]. On the other hand, RPA binds and stabilizes single-stranded DNA intermediates that form during DNA replication or upon DNA stress. Therefore, both proteins have an essential role in DNA replication and the cellular response to DNA damage [53]. Both these proteins have displayed oncogenic potential in various cancers [54,55] and were thus chosen as the proteins of interest. PRIM2 and RPA were upregulated in G1862T- compared to WT-transfected cells. DIA SWATH-based proteomics has become a well-established and unbiased technique for global proteome analysis [56]. For proteomics analysis in the current study, candidate dysregulated proteins were filtered at a q-value ≤ 0.05, absolute fold change (FC) ≥ 1.5 and a minimum of two identified unique peptides, which are acceptable metrics to limit the number of false observations. In addition, the interpretation of the proteomics data was mainly carried out in the context of functional and pathway enrichment, limiting the impact of any potential false observations. The increased expression of the two proteins of interest, PRIM2 and RPA, were validated by RT-PCR by measuring the respective RNA expression levels for both and confocal microscopy for RPA. 

In this study, transfection of Huh7 cells with HBV subgenotype A1 plasmid constructs, with or without G1862T, was used to determine the effect of this mutation on the cellular proteome. Huh7 cells are a well-differentiated, immortalized cell line derived from an HCC patient. The proteome of Huh7 cells differs from primary human hepatocytes (PHHs) or liver, and it has been suggested that caution must be exercised especially when performing drug metabolism and toxicity experiments [57]. Such experiments were not performed in the present study. Although, PHHs may be considered an ideal system, they also have several disadvantages including limited availability and lifespan, loss of hepatocyte function and susceptibility to HBV infection within days of isolation and unpredictable variability between donors [58]. Despite its limitation as an in vitro system, the Huh7 cell line provides a widely-used model that allows us to follow HBV infection and dissect complex biological processes [58,59,60] in a reproducible manner, which has been demonstrated to match findings in liver tissue and PHHs [57]. Therefore, the findings of the present study should be interpreted taking these caveats into account. Dysregulation of p38 MAPK, oxidative stress, and DNA synthesis and repair were shown in Huh7 cells transfected with G1862T compared to the wildtype. Further studies will be required using tissue and/or sera of patients infected with G1862T HBV to confirm these observations in vivo and relate them to the high hepatocarcinogenic potential of subgenotype A1 at an earlier age in SSA.

## Figures and Tables

**Table 1:** The Primers qPCR for three genes.

Gene	Direction	Sequence
PRIM2	F	5′-CTTCAGCCTCTGCTCAATCACC-3′
	R	5′-GTAACTGACGCATGCAAGGTGG-3′
RPA	F	5′-GAGCACCTATCAGCAATCCAGG-3′
	R	5′-CCTTCAGGTCTTGGACAAGCCT-3′
GAPDH	F	5′-GAAGGTGAAGGTCGGAGT-3′
	R	5′-GAAGATGGTGATGGGATTTC-3′

## Data Availability

The raw data supporting the conclusions of this article will be made available by the authors upon request.

## Supplementary Materials

The following supporting information can be downloaded at: https://www.mdpi.com/article/10.3390/cimb46070419/s1, Figure S1: HBeAg expression and viral load comparison, Figure S2: Internal validation of mass spectrometry data, Figure S3: Upregulated proteins in G1862T-transfected cells compared to WT-transfected cells, and Figure S4: Downregulated proteins in G1862T-transfected cells compared to WT-transfected cells.
