# Supplementary figures and images for: Comparison of the Proteome of Huh7 Cells Transfected with Hepatitis B Virus Subgenotype A1, with or without G1862T
Source: Curr Issues Mol Biol. 2024 Jul 4;46(7):7032–47. doi: 10.3390/cimb46070419 (PMC11275860; doi:10.3390/cimb46070419)

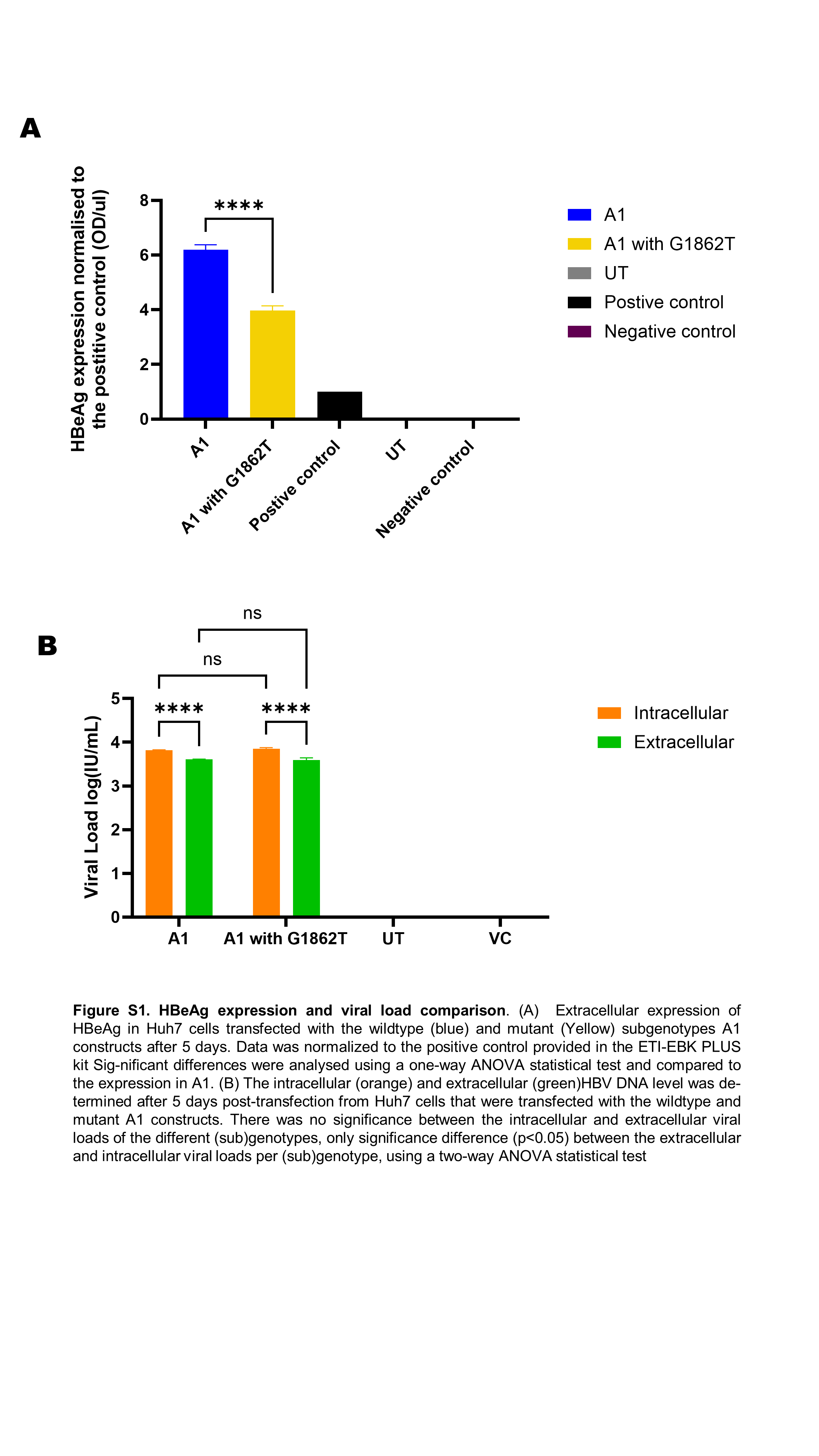

Supplement: Supplementary file 1 [file cimb-46-00419-s001.zip › S1.TIF]

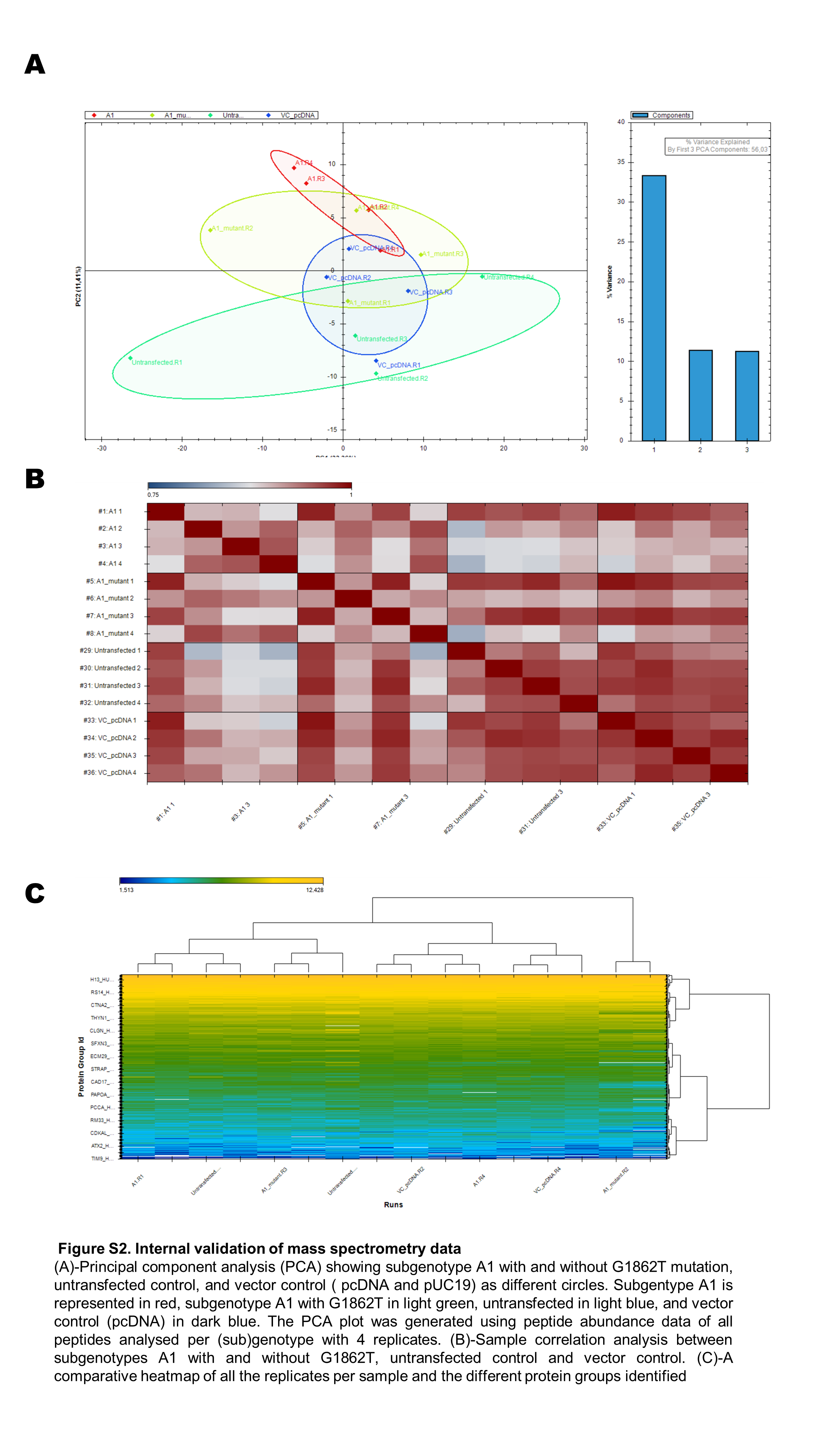

Supplement: Supplementary file 1 [file cimb-46-00419-s001.zip › S2.TIF]

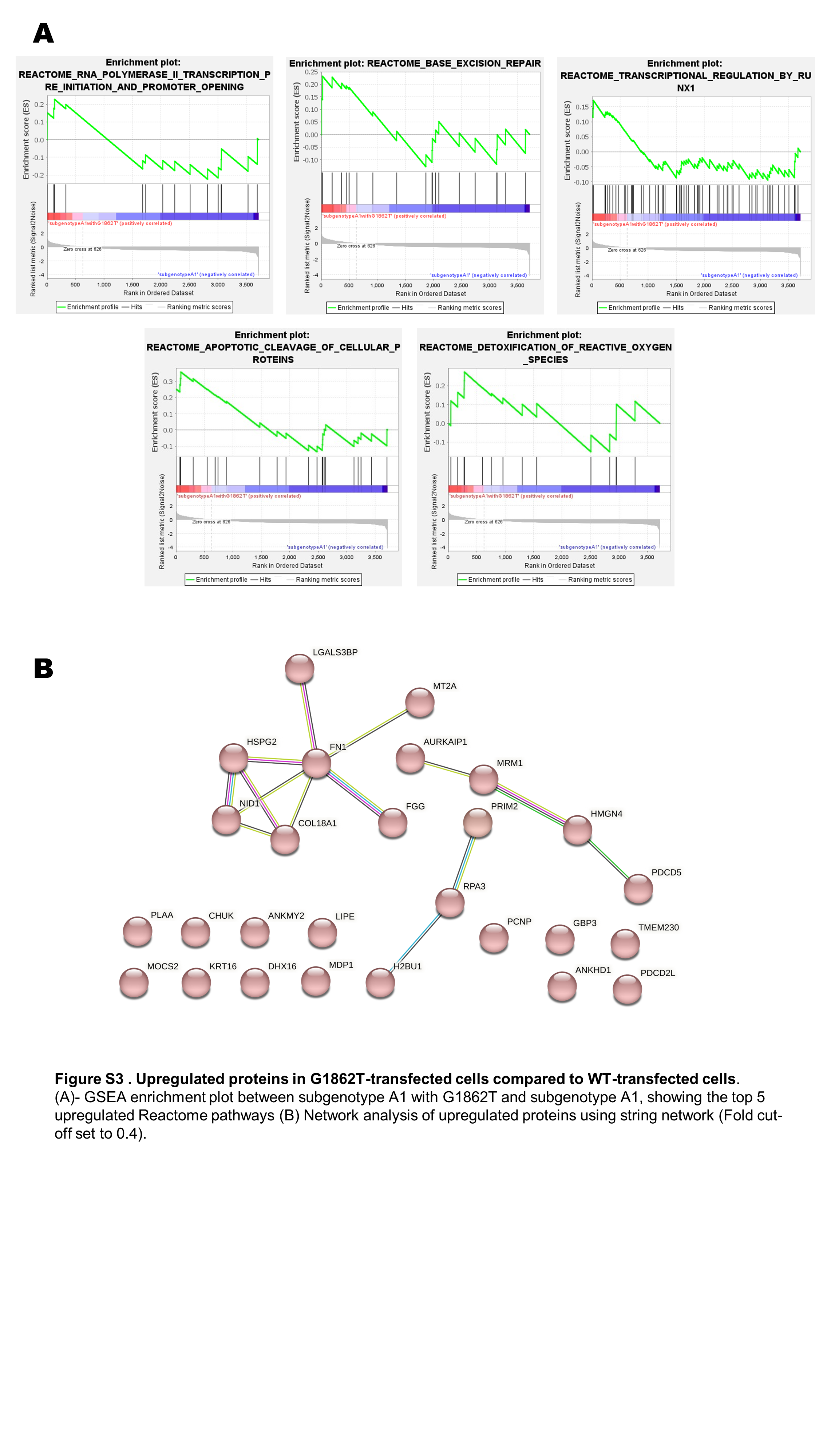

Supplement: Supplementary file 1 [file cimb-46-00419-s001.zip › S3.TIF]

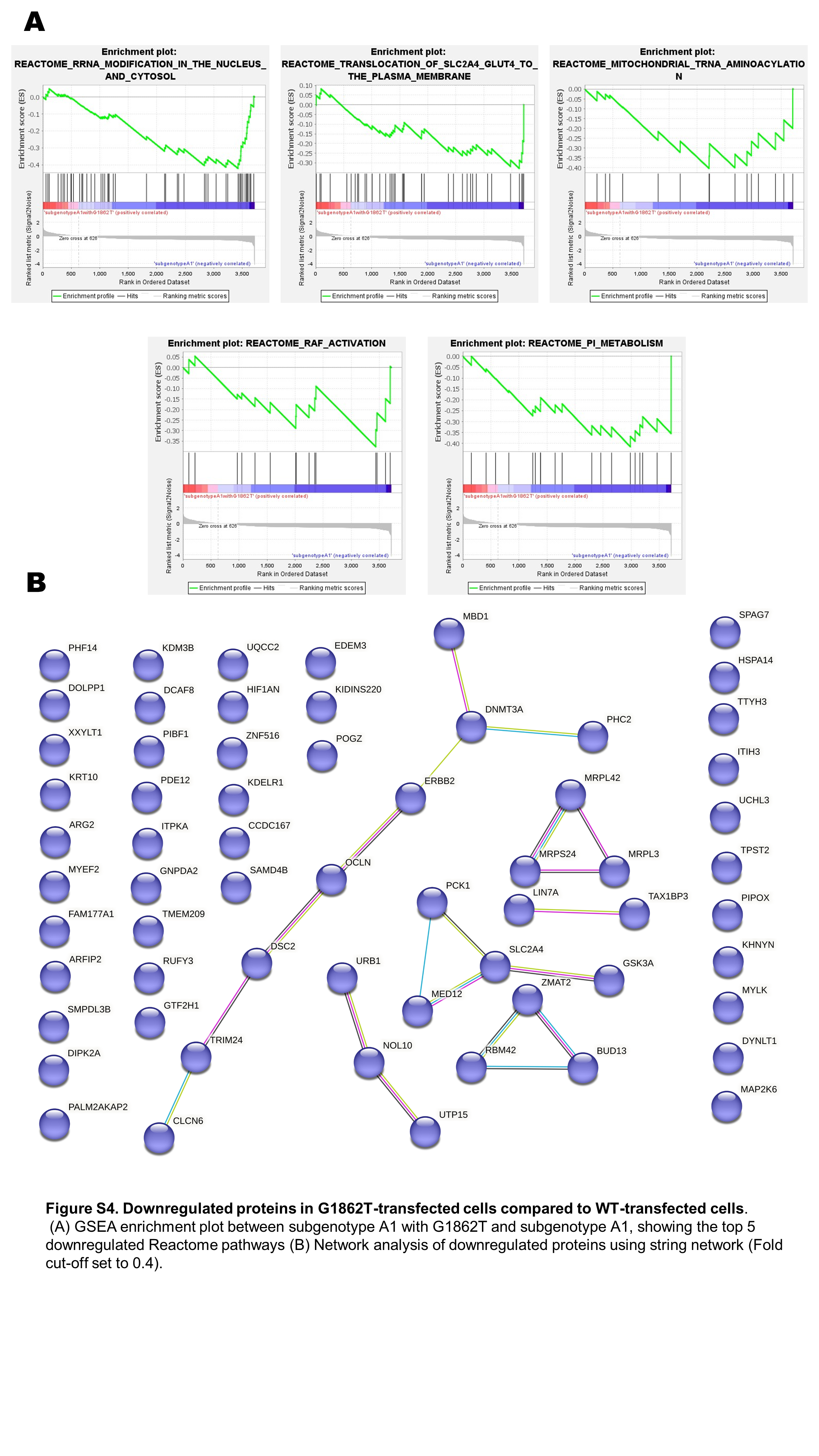

Supplement: Supplementary file 1 [file cimb-46-00419-s001.zip › S4.TIF]
